# Supplementary figures and images for: Satellite remote sensing of environmental variables can predict acoustic activity of an orthopteran assemblage
Source: PeerJ. 2022 Sep 2;10:e13969. doi: 10.7717/peerj.13969 (PMC9443809; doi:10.7717/peerj.13969)

Average dominant frequency (kHz)

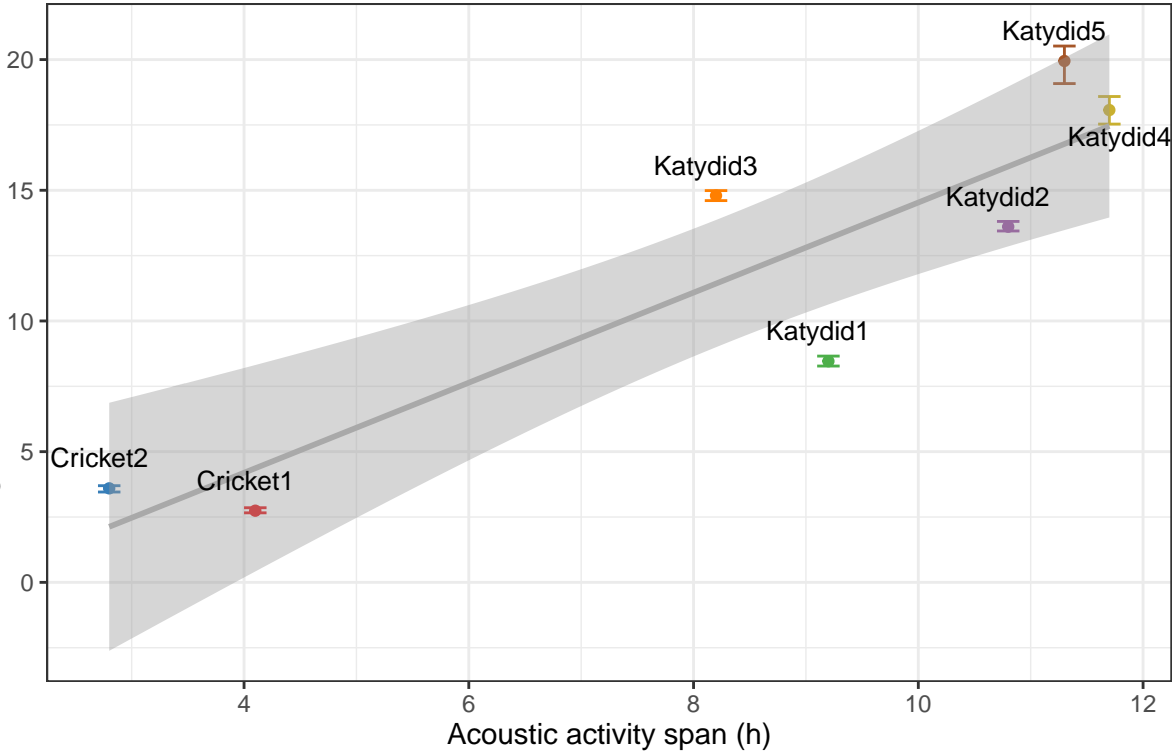

Supplement: Supplemental Information 6 — Acoustic activity span was computed from the acoustic activity distribution (Fig. 1) at a value of 0.05. Average dominant frequency error bars correspond to confidence intervals (Bootstrap 10,000 iterations), n = 5. [file peerj-10-13969-s006.pdf]
