# Supplementary material for: Satellite remote sensing of environmental variables can predict acoustic activity of an orthopteran assemblage
Source: PeerJ. 2022 Sep 2;10:e13969. doi: 10.7717/peerj.13969 (PMC9443809; doi:10.7717/peerj.13969)

**Supplementary Data S9.** Results of beta regressions for acoustic activity of seven orthopteran species in tropical Andes of Colombia.

Results top-ranked model (π = 76.2%) for detections of Sp1 (Gr2)


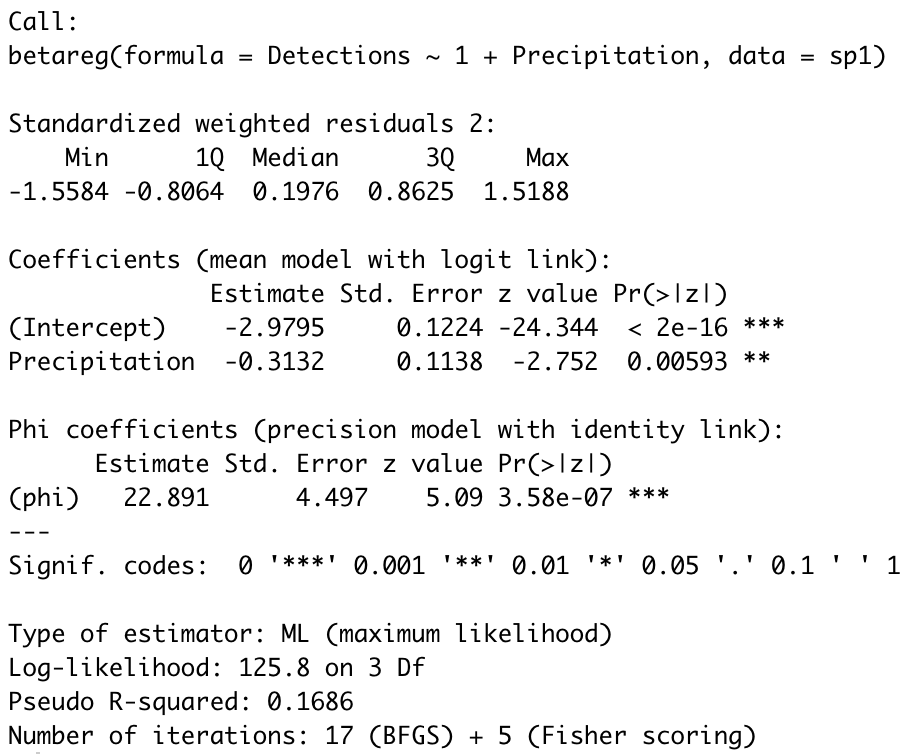


Results top-ranked model (π = 94.8%) for detections of Sp2 (Gr4)


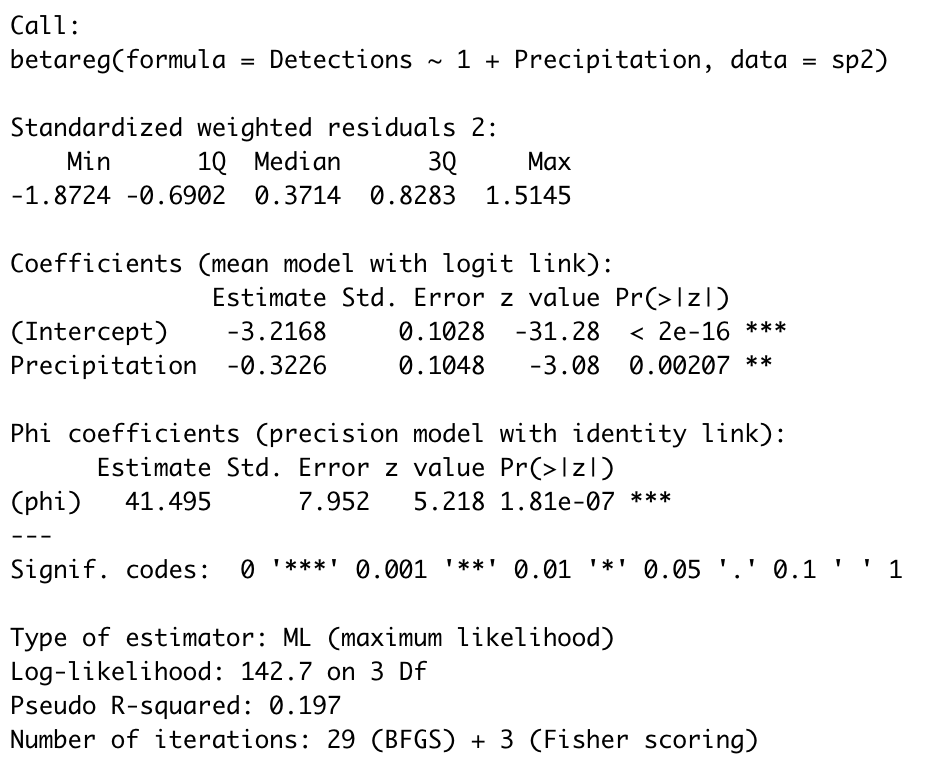


Results top-ranked model (π =82.39%) for detections of Sp3 (Gr8)


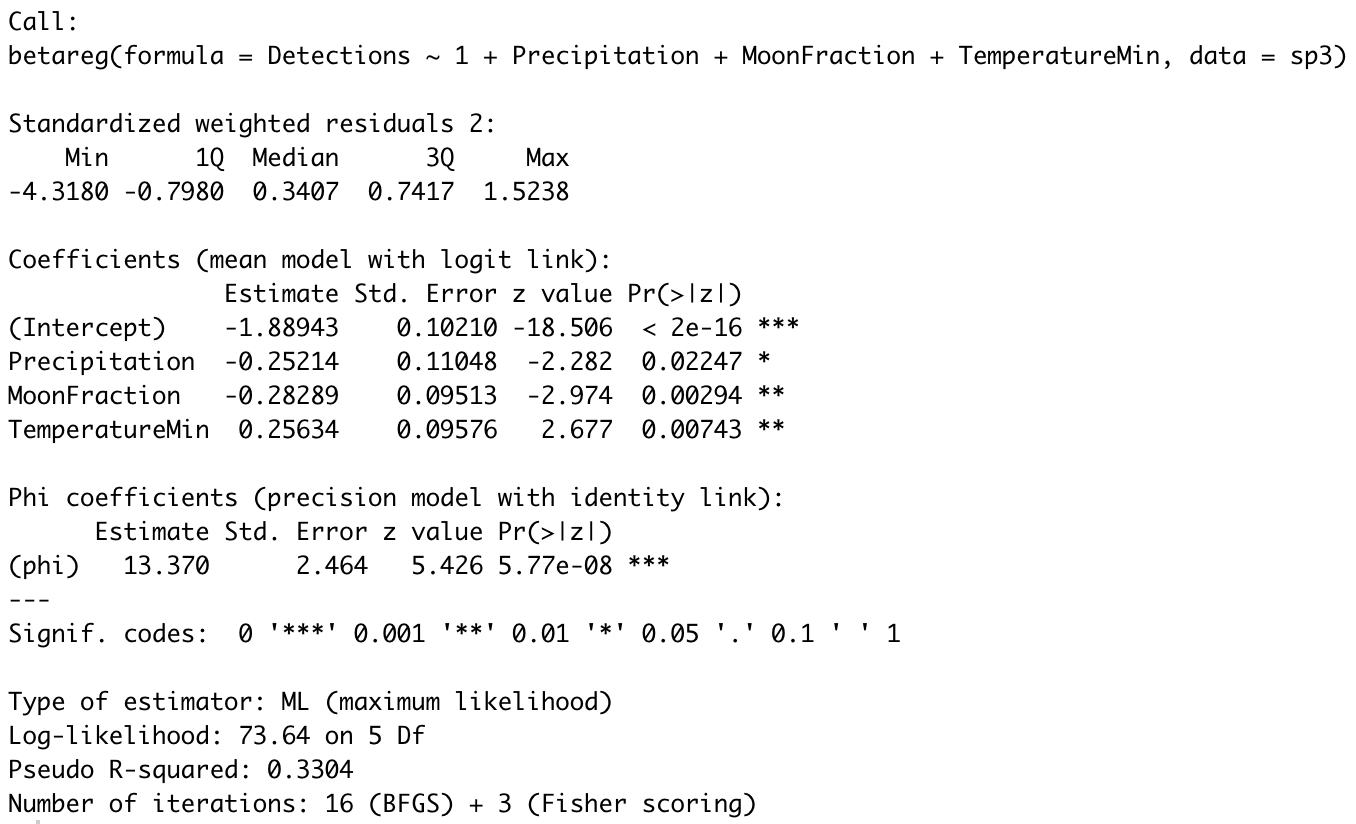


Results top-ranked model (π = 30.22%) for detections of Sp4 (Gr12)


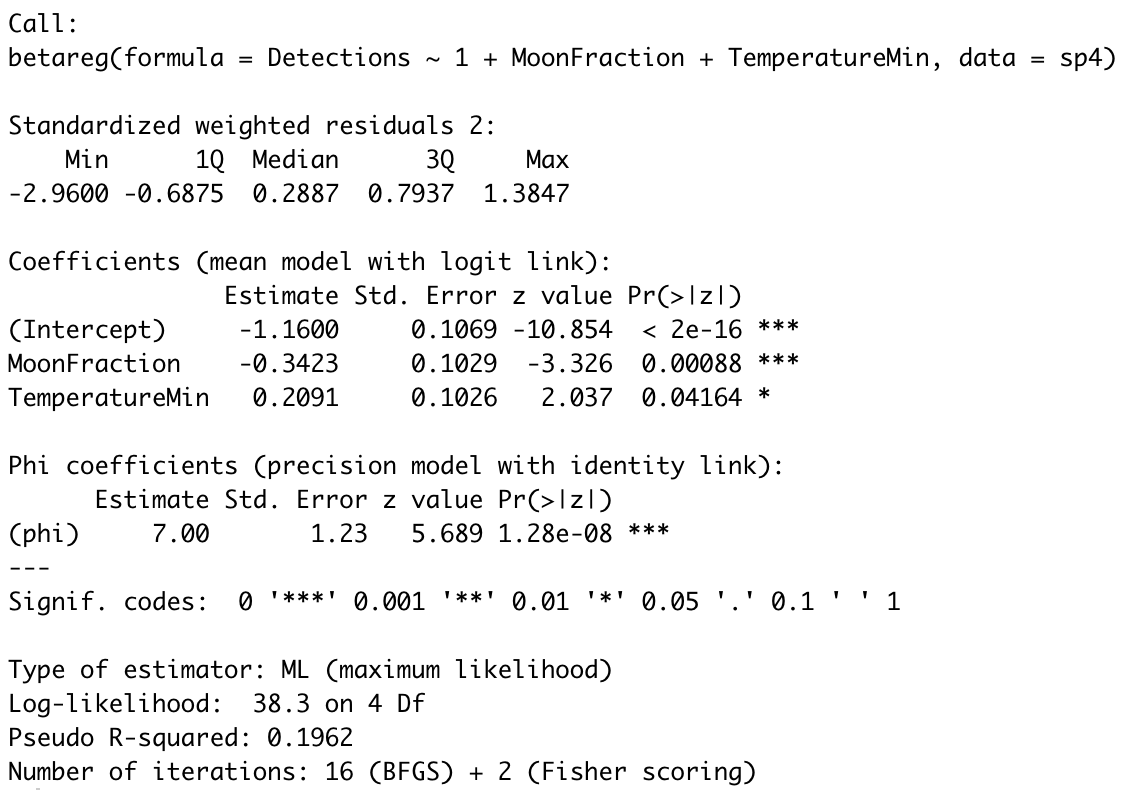


Results top-ranked model (π = 56.80%) for detections of Sp5 (Gr13)


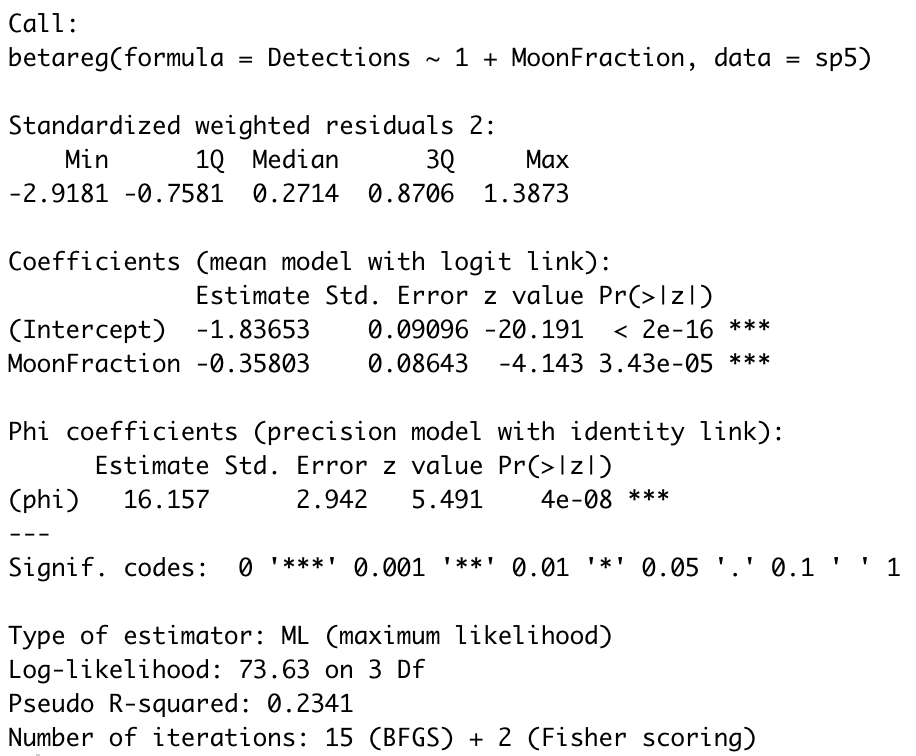


Results top-ranked model (π = 62.30%) for detections of Sp6 (Gr20)


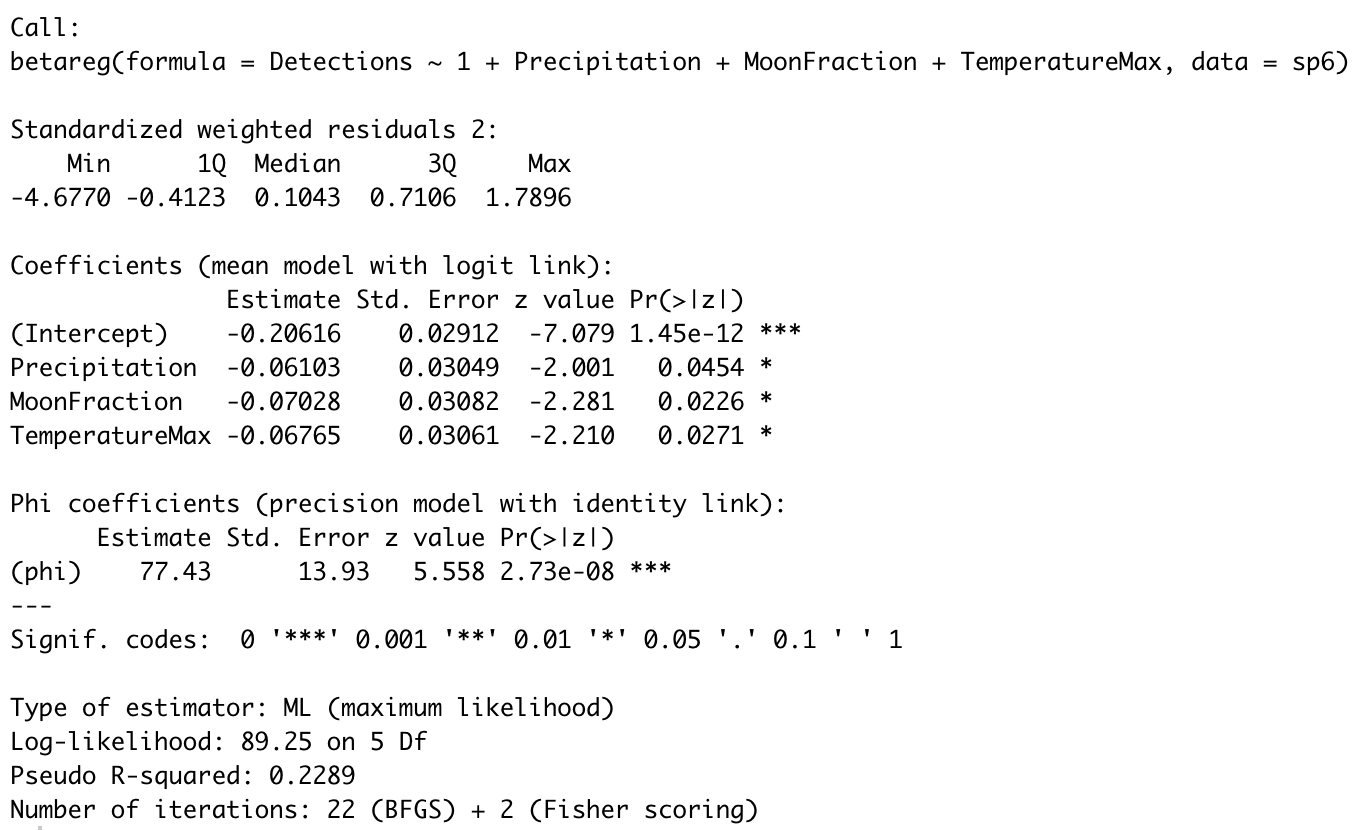


Results top-ranked model (π = 61.67%) for detections of Sp7 (Gr22)


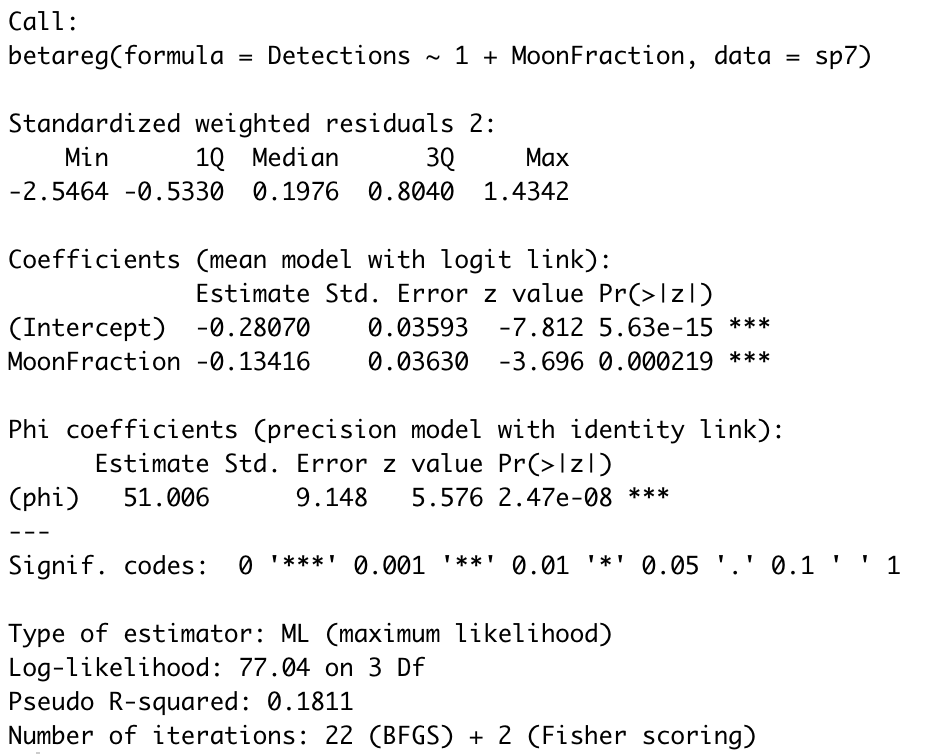

Supplement: Supplemental Information 10 [file peerj-10-13969-s010.docx]
