# Supplementary material for: Satellite remote sensing of environmental variables can predict acoustic activity of an orthopteran assemblage
Source: PeerJ. 2022 Sep 2;10:e13969. doi: 10.7717/peerj.13969 (PMC9443809; doi:10.7717/peerj.13969)
Supplement: Supplemental Information 13 — The output column indicates the output dataset included in Supplemental Data; the Model details column has a web permanent link to Rfcx Arbimon platform details. The Model column refers to the two types of models trained: RF = Radom Forests, PM = Pattern matching. Training set size is the number of recordings manually annotated to train the model (half) and test it (half). Total positive detections column is the number of recordings with presence of the species according to the model. Detection precision is the percentage value after subtracting the manually identified false positives, from the total positive detections. [file peerj-10-13969-s013.docx]

Supplemental Table 1. Model training details. The output column indicates the output dataset included in Supplemental Data; the Model details column has a web permanent link to Rfcx Arbimon platform details. The Model column refers to the two types of models trained: RF = Radom Forests, PM = Pattern matching. Training set size is the number of recordings manually annotated to train the model (half) and test it (half). Total positive detections column is the number of recordings with presence of the species according to the model. Detection precision is the percentage value after subtracting the manually identified false positives, from the total positive detections.

| Species | Model | Training set size | Training set Accuracy (%) | Training set Precision (%) | Total Positive Detections | Detection Precision (%) | Output | Online access |
| --- | --- | --- | --- | --- | --- | --- | --- | --- |
| “flutist” (Cricket1) | RF | 75 | 89 | 94 | 250 | 74 | Supplemental Data S1 | [Link](https://arbimon.rfcx.org/project/orlando-soundscapes/analysis/model/3759) |
| Podoscirtinae (Cricket2) | RF | 34 | 100 | 100 | 159 | 76 | Supplemental Data S2 | [Link](https://arbimon.rfcx.org/project/orlando-soundscapes/analysis/model/3717) |
| *Copiphora colombiae* (Katydid1) | PM | 239 | 97 | 94 | 531 | 82 | Supplemental Data S3 | [Link](https://arbimon.rfcx.org/project/orlando-soundscapes/analysis/patternmatching/5002) |
| “sprinkler” (Katydid2) | RF | 188 | 86 | 83 | 1078 | 76 | Supplemental Data S4 | [Link](https://arbimon.rfcx.org/project/orlando-soundscapes/analysis/model/3755) |
| *Neoconocephalus brachypterus* (Katydid3) | PM | 130 | 95 | 94 | 707 | 70 | Supplemental Data S5 | [Link](https://arbimon.rfcx.org/project/orlando-soundscapes/analysis/patternmatching/5014) |
| Cocconotini (Katydid4, gen. nov.) | RF | 379 | 95 | 96 | 1324 | 95 | Supplemental Data S6 | [Link](https://arbimon.rfcx.org/project/orlando-soundscapes/analysis/model/3762) |
| “rattler” (Katydid5) | PM | 296 | 98 | 99 | 1837 | 79 | Supplemental Data S7 | [Link](https://arbimon.rfcx.org/project/orlando-soundscapes/analysis/patternmatching/5016) |
