# Supplementary material for: Satellite remote sensing of environmental variables can predict acoustic activity of an orthopteran assemblage
Source: PeerJ. 2022 Sep 2;10:e13969. doi: 10.7717/peerj.13969 (PMC9443809; doi:10.7717/peerj.13969)
Supplement: Supplemental Information 14 — Total positives and False positives columns are the number of recordings for which the model detected species call presence. Detection precision is the percentage value after subtracting false positives from total positives. [file peerj-10-13969-s014.docx]

Supplemental Table S2. Manual revision of false positives. Total positives and False positives columns are the number of recordings for which the model detected species call presence. Detection precision is the percentage value after subtracting false positives from total positives.

| Species | Total positives | False Positives | Detection Precision (%) |
| --- | --- | --- | --- |
| Cricket1 | 250 | 87 | 74 |
| Cricket2 | 159 | 51 | 76 |
| Katydid1 | 531 | 118 | 82 |
| Katydid2 | 1078 | 338 | 76 |
| Katydid3 | 707 | 296 | 70 |
| Katydid4 | 1324 | 64 | 95 |
| Katydid5 | 1837 | 481 | 79 |
