# Supplementary material for: Satellite remote sensing of environmental variables can predict acoustic activity of an orthopteran assemblage
Source: PeerJ. 2022 Sep 2;10:e13969. doi: 10.7717/peerj.13969 (PMC9443809; doi:10.7717/peerj.13969)
Supplement: Supplemental Information 15 — Values in parenthesis correspond to confidence intervals (Bootstrap 10,000 iterations). n = 5 measurements. [file peerj-10-13969-s015.docx]

Supplemental Table S3. Average maximum, minimum, and dominant frequencies per species measured from the recordings in Supplemental Data S9. Values in parenthesis correspond to confidence intervals (Bootstrap 10000 iterations). n = 5 measurements.

| species | Min. Frequency (kHz) | Max. Frequency (kHz) | Dom. Frequency (kHz) |
| --- | --- | --- | --- |
| Cricket1 | 2.626 (2.554-2.721) | 2.863 (2.778-2.973) | 2.742 (2.660-2.855) |
| Cricket2 | 3.428 (3.299-3.523) | 3.723 (3.580-3.823) | 3.598 (3.458-3.698) |
| Katydid1 | 7.661 (7.494-7.842) | 9.428 (9.267-9.588) | 8.460 (8.276-8.656) |
| Katydid2 | 12.733 (12.534-12.946) | 14.027 (13.697-14.362) | 13.606 (13.443-13.808) |
| Katydid3 | 13.511 (13.350-13.680) | 15.694 (15.475-15.922) | 14.797 (14.605-14.987) |
| Katydid4 | 16.722 (16.272- 17.181) | 19.812 (19.349-20.250) | 18.069 (17.532-18.587) |
| Katydid5 | 18.215 (15.942-19.672) | 20.933 (20.008-21.554) | 19.946 (19.081-20.518) |
