# Supplementary material for: Satellite remote sensing of environmental variables can predict acoustic activity of an orthopteran assemblage
Source: PeerJ. 2022 Sep 2;10:e13969. doi: 10.7717/peerj.13969 (PMC9443809; doi:10.7717/peerj.13969)
Supplement: Supplemental Information 16 [file peerj-10-13969-s016.docx]

El Monitoreo Acústico Pasivo (PAM en inglés) es un método de evalución de la biodiversidad que permite muestreos más extensos y menos intrusivos que otros métodos tradicionales (p. e., colecta de especímenes), al usar grabaciones de audio como fuente de información. Los insectos tienen un gran potencial como modelos para el estudio y monitoreo de ensamblajes acústicos debido a su sensibilidad a cambios ambientales. Sin embargo, los estudios de ecoacústica enfocados en insectos aún son escasos si se comparan con grupos más carismáticos. Los patrones de actividad de los insectos responden a factores ambientales como temperatura, luz de luna, o precipitación; a pesar de ello, perspectivas de comunidad acústica aún han sido poco exploradas. En este trabajo presentamos un ejemplo de la utilidad de PAM para monitoreo de patrones temporales de actividad en un ensamblaje de insectos nocturnos (Orthoptera). Integramos medidas de factores ambientales de sensores remotos y estimaciones astronómicas a escala local en un Bosque Andino de Colombia y evaluamos la respuesta acústica de los ortópteros a través de detecciones automáticas de sus cantos durante nueve semanas (marzo y abril de 2020). Describimos el rango de frecuencia y la actividad acústica diaria de cada especie representativa. Tres especies se solaparon en frecuencia y actividad diaria, pero habitan diferentes estratos: dosel, sotobosque y a nivel del suelo. Según la frecuencia y actividad acústica, identificamos tres tendencias: i) las dos especies de grillos cantan a menores frecuencias, por períodos de tiempo más cortos (anochecer); ii) las especies de esperanzas cantan a frecuencias mas altas, y por períodos de tiempo más largos, a lo largo de la noche; y iii) la ventana de actividad acústica diaria parece aumentar proporcionalmente con la frecuencia acústica dominante, sin embargo, se requieren estudios más detallados en este aspecto. También identificamos un coro del anochecer en el cual todas las especies cantan al mismo tiempo. Para cuantificar la respuesta a factores ambientales, calculamos una regresión beta con la actividad acústica como variable respuesta de luz de luna, temperatura superficial, y precipitación como variables explicativas. La respuesta a luz de luna fue significativa para las esperanzas, pero no para los grillos, debido posiblemente a las diferencias en actividad acústica diaria. Los grillos son activos durante el anochecer solamente, así que los efectos de la luz de luna son insignificantes. La respuesta a precipitación fue significativa para los dos grillos, pero no para las esperanzas; debido posiblemente a la mayor probabilidad de que la lluvia interrumpa las ventanas de los grillos, al ser menos extensas. Nuestro estudio muestra como el muestreo de ensamblajes acústicos de ortópteros a resolución taxonómica de especie acoplado con mediciones de sensores remotos puede revelar respuestas a factores ambientales. Adicionalmente, demostramos como la información satelital puede ser una fuente alternativa de datos ambientales para estudios a nivel de comunidad con limitaciones geográficas, financieras, u otras.
