# Supplementary material for: Satellite remote sensing of environmental variables can predict acoustic activity of an orthopteran assemblage
Source: PeerJ. 2022 Sep 2;10:e13969. doi: 10.7717/peerj.13969 (PMC9443809; doi:10.7717/peerj.13969)
Supplement: Supplemental Information 17 — This translation was not reviewed by PeerJ staff or the Board and is the authors' own rendering of the article. [file peerj-10-13969-s017.pdf]

# Teledetección de variables ambientales por satélite puede predecir la actividad acústica de un ensamblaje de ortópteros

Diego A. Gomez-Morales[1,2] , Orlando Acevedo-Charry[3,4]

[1] Departamento de Biología, Universidad Nacional de Colombia, Bogotá, D.C., Colombia.
[2] Department of Biology, California State University, Northridge, CA, USA
[3] Colección de Sonidos Ambientales *Mauricio Álvarez-Rebolledo*, Colecciones Biológicas, Subdirección de Investigaciones, Instituto de Investigación de Recursos Biológicos Alexander von Humboldt, Villa de Leyva, Boyacá, Colombia
[4] School of Natural Resources and Environment, Department of Wildlife Ecology and Conservation, & Florida Museum of Natural History, University of Florida, Gainesville, FL, USA

Correspondencia:
Diego A. Gomez-Morales[1]
Department of Biology, California State University Northridge, 18111 Nordhoff Street, Northridge, CA, 91330-8303, USA
Email address: diego.gomez.519@my.csun.edu

## Resumen

El Monitoreo Acústico Pasivo (PAM en inglés) es un método de evalución de la biodiversidad que permite muestreos más extensos y menos intrusivos que otros métodos tradicionales (p. e., colecta de especímenes), al usar grabaciones de audio como fuente de información. Los insectos tienen un gran potencial como modelos para el estudio y monitoreo de ensamblajes acústicos debido a su sensibilidad a cambios ambientales. Sin embargo, los estudios de ecoacústica enfocados en insectos aún son escasos si se comparan con grupos más carismáticos. Los patrones de actividad de los insectos responden a factores ambientales como temperatura, luz de luna, o precipitación; a pesar de ello, perspectivas de comunidad acústica aún han sido poco exploradas. En este trabajo presentamos un ejemplo de la utilidad de PAM para monitoreo de patrones temporales de actividad en un ensamblaje de insectos nocturnos (Orthoptera). Integramos medidas de factores ambientales de sensores remotos y estimaciones astronómicas a escala local en un Bosque Andino de Colombia y evaluamos la respuesta acústica de los ortópteros a través de detecciones automáticas de sus cantos durante nueve semanas (marzo y abril de 2020). Describimos el rango de frecuencia y la actividad acústica diaria de cada especie representativa. Tres especies se solaparon en frecuencia y actividad diaria, pero habitan diferentes estratos: dosel, sotobosque y a nivel del suelo. Según la frecuencia y actividad acústica, identificamos tres tendencias: i) las dos especies de grillos cantan a menores frecuencias, por períodos de tiempo más cortos (anochecer); ii) las especies

de esperanzas cantan a frecuencias más altas, y por períodos de tiempo más largos, a lo largo de la noche; y iii) la ventana de actividad acústica diaria parece aumentar proporcionalmente con la frecuencia acústica dominante, sin embargo, se requieren estudios más detallados en este aspecto. También identificamos un coro del anochecer en el cual todas las especies cantan al mismo tiempo. Para cuantificar la respuesta a factores ambientales, calculamos una regresión beta con la actividad acústica como variable respuesta de luz de luna, temperatura superficial, y precipitación como variables explicativas. La respuesta a luz de luna fue significativa para las esperanzas, pero no para los grillos, debido posiblemente a las diferencias en actividad acústica diaria. Los grillos son activos durante el anochecer solamente, así que los efectos de la luz de luna son insignificantes. La respuesta a precipitación fue significativa para los dos grillos, pero no para las esperanzas; debido posiblemente a la mayor probabilidad de que la lluvia interrumpa las ventanas de los grillos, al ser menos extensas. Nuestro estudio muestra como el muestreo de ensamblajes acústicos de ortópteros a resolución taxonómica de especie acoplado con mediciones de sensores remotos puede revelar respuestas a factores ambientales. Adicionalmente, demostramos como la información satelital puede ser una fuente alternativa de datos ambientales para estudios a nivel de comunidad con limitaciones geográficas, financieras, u otras.

## Introducción

Muchos animales utilizan la señalización acústica como su principal forma de comunicación (Bradbury & Vehrencamp, 2011), así contribuyendo al componente biótico del paisaje sonoro (Pijanowski et al. 2011). Las preguntas ecológicas sobre el comportamiento, la actividad estacional o la respuesta a factores externos de los animales a diferentes escalas ecológicas y temporales de las comunidades acústicas pueden abordarse con el monitoreo acústico (Sugai et al., 2019; Gottesman et al., 2021; Chhaya et al., 2021). Este puede realizarse con grabación directa en persona, en campo (Drewry & Rand, 1983; Diwakar & Balakrishnan, 2007a), o con grabación automática pasiva (Deichmann et al., 2018). El Monitoreo Acústico Pasivo (PAM) se ha convertido en uno de los métodos más populares para obtener grabaciones ambientales gracias al creciente acceso a las nuevas tecnologías de grabación e incremento en potencia de cálculo (Riede, 2018; Sugai et al., 2019). El PAM consiste en el despliegue de grabadoras autónomas pasivas sobre el terreno. Este implica ventajas cuando se usa para el monitoreo de la biodiversidad como períodos de evaluación más largos, métodos menos intrusivos, mayor recolección de datos y mayor potencial para la investigación bioacústica de comunidades a diferentes escalas, si se compara con enfoques clásicos como la recolección de especímenes en campo (Blumstein et al., 2011; Deichmann et al., 2018; Sugai et al., 2020). Además, PAM permite clasificar los cantos de llamado en unidades taxonómicas reconocibles, también denominadas morfoespecies acústicas o sonotipos (Riede, 1998; Aide et al., 2013; Ferreira et al., 2018). A pesar de ello, sigue habiendo problemas cuando se aplica a grupos taxonómicos muy diversos con poca disponibilidad de descripciones taxonómicas y acústicas, como los insectos (Riede, 2018). Para abordar estos retos, monitoreamos acústicamente un ensamblaje de

ortópteros nocturnos y asignamos los sonotipos a sus respectivas especies taxonómicas identificadas a partir de especímenes capturados en el sitio.

Los sonidos de los insectos impulsan los paisajes sonoros tropicales con sus llamados que varían en tiempo, frecuencia acústica y escalas espaciales (Aide et al., 2017). Además, los sonidos de los insectos tienen un alto potencial como bioindicadores acústicos, especialmente a escala local, debido a su sensibilidad al cambio ambiental (McGeoch, 2007; Jeliazkov et al., 2016; Riede, 2018). Dentro de los insectos, los grillos y las esperanzas (Orthoptera: Ensifera) son algunos de los contribuyentes acústicos más importantes de los paisajes sonoros. Producen sonidos frotando unas estructuras especializadas de sus alas, un comportamiento denominado estridulación elitral (Baker & Chesmore, 2020). Entre los distintos tipos de llamados estridulatorios, el "canto de llamada" es utilizado por los machos para atraer al sexo opuesto (Grimaldi & Engel, 2005; Buellesbach, Cash & Schmitt, 2018). Los cantos de llamado son los más comunes en los paisajes sonoros naturales, y se utilizan a menudo en los estudios taxonómicos y evolutivos (Tan et al., 2021) debido a sus características estereotipadas y a su gran especificidad especie específica. (Grimaldi & Engel, 2005). Sin embargo, pocos estudios de ecoacústica incluyen una resolución taxonómica detallada de ortópteros u otros insectos (Diwakar & Balakrishnan, 2007a; Gasc et al., 2018) y muchos enfoques a escala de comunidad se enfocan más en vertebrados carismáticos como aves (Tobias et al., 2014), anuros (Villanueva-Rivera, 2014) o mamíferos (Heinicke et al., 2015). Si hablamos de estudios a escala de comunidad, el asignar las especies acústicas a las taxonómicas ayudará a desvelar la estructura y el cambio de las comunidades biológicas en espacio y tiempo (Chhaya et al., 2021).

A nivel de comunidad, los cambios en los patrones de actividad acústica pueden explicarse por la influencia de un conjunto de factores bióticos y abióticos (Chhaya et al., 2021). Las explicaciones de cómo los factores bióticos influyen en los patrones acústicos han sido formalizadas por varias teorías (Farina, 2014). Entre ellas por ejemplo, la hipótesis del nicho acústico afirma que los animales responden a otros emisores al particionar su actividad acústica (Krause, 1993) para evitar el enmascaramiento acústico (o solapamiento) en tiempo, frecuencia espectral y espacio; optimizando así la transmisión de la señal (Schmidt, Römer y Riede, 2013). Sin embargo, el solapamiento temporal es habitual en los ensamblajes de ortópteros tropicales, especialmente durante el coro del atardecer (Riede, 1996; Jain et al., 2014). La tendencia de los animales de diferentes especies a concentrar su canto en la misma ventana temporal se ha denominado anteriormente agrupamiento o “clustering” en inglés (Tobias et al., 2014) y puede beneficiar a los emisores individuales al reducir los riesgos de depredación. (Jain et al., 2014; Farina & Ceraulo, 2017). Por otro lado, se sabe que los factores ambientales abióticos, como la temperatura, las precipitaciones o la luz de luna pueden predecir los patrones acústicos de las comunidades de vertebrados (Bruni, Mennill & Foote, 2014; Pérez-Granados, Schuchmann & Marques, 2022), así como en las de insectos (Lang et al., 2006; Franklin et al., 2009). La actividad acústica puede ser una descripción muy útil de los patrones circadianos y estacionales

(Aide et al., 2013), y de las respuestas de las especies a las variables ambientales (Pérez-Granados, Schuchmann & Marques, 2022).

La mayoría de los estudios a escala de comunidad recogen la información ambiental a nivel local, midiendo las variables directamente en campo, o recopilándola de las estaciones meteorológicas locales (Ospina et al., 2013). Sin embargo, la información ambiental de campo podría ser difícil o incluso imposible de obtener en algunas circunstancias. Por ejemplo, debido a la escasez o ausencia de estaciones meteorológicas en algunas áreas, la ubicación remota de los sitios de estudio, o las limitaciones de financiación. La teledetección por satélite podría ser una alternativa en estos casos, ya que es exhaustiva espaciotemporalmente, a pesar de su menor resolución. Los datos recogidos mediante teledetección por satélite han sido útiles para estudios ecológicos a nivel de paisaje o de hábitat (Pettorelli et al., 2014; Pasetto et al., 2018) pero su potencial para preguntas a escala de comunidad, como la relación con la actividad acústica, sigue sin explorarse.

En este trabajo caracterizamos la actividad acústica de un ensamblaje de ortópteros, midiendo la ocurrencia de eventos de canto por unidad de tiempo y el nivel de solapamiento interespecífico en el rango temporal y de frecuencia. En principio esperamos encontrar un grupo de especies cantantes durante el crepúsculo (coro del crepúsculo), pero ninguna especie que se solapara en las tres dimensiones (tiempo, frecuencia y espacio) con otra. También decidimos explorar la implementación de datos de teledetección satelital de variables ambientales (temperatura superficial, precipitación diaria) junto con la fracción iluminada de la luna calculada astronómicamente, para describir su efecto especie específico en la actividad acústica de un ensamblaje de ortópteros en los Andes tropicales de Colombia. Adicionalmente, grabamos y recolectamos los insectos (incluyendo algunas especies nuevas para la ciencia que están siendo descritas) para identificar los sonotipos a nivel taxonómico de especie (cuando posible). Este trabajo sirve como una base importante para futuros protocolos de monitoreo acústico de insectos mediante la identificación de bioindicadores acústicos y el uso de datos de teledetección como una fuente de información alternativa para estudios localizados de ecología de comunidades.

## Materiales y métodos

*Lugar del estudio y muestreo acústico*

Muestreamos el paisaje sonoro en la Reserva Natural Los Tucanes, ubicada en Gachantivá, Boyacá, Andes Orientales de Colombia (5.789, -73.550; 2300 ± 25msnm). Esta reserva privada incluye un área de 0,16 $km^2$ de bosque subandino dominado por roble andino (*Quercus humboldtii*) en diferentes estados de regeneración, con una temperatura anual de 15°C. La precipitación anual es de unos 1450 mm, en dos épocas de lluvia: de marzo a mayo y de octubre a noviembre (Climate-Data.org, 2019). Es mayoritariamente un bosque secundario de 20 años de

edad con pastizales de sucesión temprana tras el abandono agrícola. Utilizamos los datos de monitoreo acústico de una grabadora Audiomoth 1.0.0 desplegada a 1,5m del suelo, a una tasa de muestreo de 48kHz y resolución de 16 bits, configurada para grabar 1 minuto cada 30 minutos durante la temporada de lluvias, de marzo a mayo de 2020 (Tovar García & Acevedo-Charry, 2021).

El conjunto de grabaciones (n=2851) se normalizó a -3dB y se subió a la plataforma en línea ARBIMON de Rainforest Connection-RFCx (arbimon.rfcx.org)(información de descarga disponible en Supplemental Data 11). A partir de ahí, exploramos manualmente una submuestra de grabaciones (Supplemental Table S1, training set size) para anotar la presencia o ausencia de las especies acústicas observadas consistentemenete a lo largo de los días. Definimos los sonotipos antes de identificar las especies taxonómicas (véase más adelante) que se utilizaron durante el análisis de los datos. Otros sonotipos con actividad acústica esporádica no se consideraron en los análisis, dada la dificultad de entrenar modelos de detección a partir de muy pocas grabaciones anotadas. Después entrenamos modelos de reconocimiento automático de bosques aleatorios (Supplemental Data S1, S2, S4, S6) y de "pattern matching" (o coincidencia de patrones) (Supplemental Data S3, S5, S7) utilizando las herramientas integradas de RFCx ARBIMON para cada sonotipo, con las grabaciones anotadas como conjunto de datos de entrenamiento (Supplemental Table 1) con el fin de detectar las ocurrencias de los sonotipos para cada grabación. Determinamos que el rendimiento de los modelos de coincidencia de patrones fue mejor para las esperanzas, mientras que los modelos de bosques aleatorios fueron significativamente mejores para los grillos, según nuestras pruebas preliminares. Una comparación detallada del rendimiento entre los modelos de detección disponibles (incluidos aquellos dirigidos a otros grupos animales) para las especies de ortópteros es un tema interesante para abordar en estudios futuros, más cuando ahora contamos con nuevas herramientas prometedoras (por ejemplo, Lapp et al., 2021; Steinfath et al., 2021). Los resultados de los modelos se revisaron manualmente, se descartaron los falsos positivos y se calculó la precisión de la detección como se sugiere en Aide et al. (2013). Todos los modelos de detección presentaron una precisión superior al 70% (Supplemental Table S2). Después de la post validación, la salida del modelo fue una matriz de presencias/ausencias por grabación para el conjunto completo. Definimos la actividad acústica como las ocurrencias detectadas por unidad de tiempo: horas y días (Aide et al., 2013). Partiendo de la actividad acústica (ocurrencias) por hora, describimos la actividad diaria promedio por especie, y medimos la partición temporal entre pares de especies usando el método de solapamiento de densidades de kernel, que es un coeficiente que refleja el grado de solapamiento entre los patrones de actividad como medida de similitud (Ridout & Linkie, 2009). Además, utilizamos la actividad acústica por día para ajustar modelos de respuesta a variables ambientales teledetectadas por satélite (véase más adelante).

Para asignar los sonotipos con su identidad taxonómica, recogimos ejemplares en el campo y realizamos la delimitación taxonómica buscando activamente los emisores de cada señal de los

sonotipos seleccionados (los activos consistentemente en el conjunto de datos PAM) durante dos salidas de campo, en septiembre y octubre de 2020. Durante estas salidas de campo se realizaron observaciones del microhábitat (altura) y del comportamiento de canto. Los especímenes recolectados fueron depositados en la colección entomológica del Instituto Humboldt (IAvH-E) en Villa de Leyva (Boyacá) siguiendo las pautas para especímenes extendidos (Acevedo-Charry et al., 2021) con un voucher genético (pata delantera) también depositado en la colección de tejidos del Instituto Humboldt en Palmira (Valle del Cauca, Colombia). Los especímenes fueron identificados a la mayor resolución taxonómica posible. No pudimos recolectar especímenes de algunas especies (habitantes del dosel), por lo que asignamos un nombre temporal de sonotipo (por ejemplo, "Flutist") a la señal acústica (Aide et al., 2017; Ferreira et al., 2018). Además, realizamos grabaciones de algunos individuos en cautiverio (especies: Katydid1, Katydid3, Katydid4) o directamente en el campo (Katydid5) con una grabadora Audiomoth 1.0.0 a una frecuencia de muestreo de 384 kHz y una resolución de 16 bits. Las grabaciones de referencia para otras especies (Cricket1, Cricket2 y Katydid2) se realizaron en campo con un micrófono shotgun Sennheiser ME67 conectado a una grabadora SoundDevice Mix Pre 3 de primera generación. Las grabaciones de referencia se depositaron en la Colección de Sonidos Ambientales *Mauricio Álvarez-Rebolledo* del Instituto Humboldt (IAvH-CSA-18783 a IAvH-CSA-18805).

*Variables ambientales a partir de datos de teledetección*

En las fechas de detección a lo largo de nuestro tiempo de estudio, evaluamos la relación de la actividad acústica por día de cada especie con la temperatura local y la precipitación detectada por satélite, y la luz de la luna calculada astronómicamente. Extrajimos las series temporales del píxel superpuesto al punto de muestreo a partir de archivos ráster generados diariamente utilizando el software Quantum GIS (Equipo de desarrollo de QGIS, 2022). La temperatura se obtuvo a partir de un conjunto de datos de 1 km de resolución utilizando el parámetro de temperatura de superficie terrestre ("Land Surface Temperature", LST) generado con los productos LST de espectrorradiómetro de imágenes de resolución moderada (MODIS en inglés) (Zhang et al., 2021, 2022); este conjunto de datos incluye una medición a las 01:00 y otra a las 13:00 horas, que equivale a la temperatura mínima y máxima diaria. Por otro lado, los valores de precipitación se obtuvieron a partir de un conjunto de datos de 10 km de resolución estimados mediante el Integrated Multi-satellitE Retrievals for Global Precipitation Measurement (IMERG) (Huffman et al., 2022). Los datos ambientales utilizados para el análisis se proporcionan en Supplemental Data S8. Análisis preliminares mostraron una alta correlación con la estación meteorológica nacional más cercana en Santa Sofía, Boyacá (a 10 km de distancia de nuestro sitio de estudio) pero los datos de IMERG incluyen valores más estables (es decir, sin mediciones faltantes en algunos días, como tenía Santa Sofía). Por último, la fracción iluminada de la luna se obtuvo a través de la función *getMoonIllumination* del paquete R *suncalc* para las coordenadas de nuestro sitio de muestreo (Thieurmel & Elmarhraoui, 2019).

*Análisis de datos*

Para las variables temporales, se analizó la actividad acústica diaria de cada especie utilizando el paquete *overlap* en *R* (Ridout & Linkie, 2009). Primero, generamos una distribución de densidad del kernel de von Mises, de la actividad acústica diaria por cada especie durante el periodo completo de muestreo (marzo a abril), dado que los patrones de actividad acústica diaria corresponden a una distribución circular. A continuación, calculamos el coeficiente delta ($\hat{\Delta}_4$) de solapamiento entre cada par de especies, tal y como se recomienda para muestras mayores a 75 (Ridout & Linkie, 2009) con el valor de suavizado por defecto. Los intervalos de confianza se calcularon utilizando un bootstrap suavizado con 10.000 muestras, ajustado para sesgo (Ridout & Linkie, 2009).

Las variables espectrales, es decir, la frecuencia dominante (frecuencia con la mayor energía), el ancho de banda de frecuencia desde la mínima (-5dB por debajo de la frecuencia dominante) a la máxima (+5dB por encima de la frecuencia dominante), se midieron manualmente en la ventana de análisis FFT (ventana Hamming, 256 muestras) del software *ocenaudio* (Equipo de ocenaudio, 2015) para el armónico dominante de dieciocho sílabas de canto, como se define en (Baker & Chesmore, 2020) para las especies de eperanzas, y dieciocho segmentos de 1s para las especies de grillos. Los segmentos se seleccionaron de las grabaciones con menos ruido del conjunto de datos PAM para cada especie (Supplemental Data S10). No se midieron los armónicos adicionales debido a que la grabadora no los detectó constantemente por la atenuación (Romer & Lewald, 1992; Hung & Prestwich, 2004). Para evaluar el solapamiento de frecuencias, contrastamos las mediciones (frecuencias mínimas, máximas y dominantes) para cada especie al remuestrar aleatoriamente las mediciones por 10.000 iteraciones. Cada iteración por especie y medición incluye un valor promedio e intervalos de confianza del 95% de los datos (Supplemental Table S3).

Exploramos la relación de la proporción de detecciones de cada especie por día con las cuatro variables continuas de teledetección mediante una regresión beta, utilizando el paquete *betareg* en R (Cribari-Neto & Zeileis, 2010). Asumimos una distribución beta porque los valores son continuos y limitados entre 0 y 1 (Bolker, 2007), escalamos las ocurrencias con la fórmula $Detecciones_{(b)} = [Detecciones_{(a)} * (N - 1) + 0,5]/N$, donde $N$ es el tamaño de la muestra y $Detecciones_{(b)}$ es nuestra variable respuesta para la regresión beta (Smithson & Verkuilen, 2006). Para seleccionar la variable que mejor se ajusta a la variable de respuesta (proporción de detecciones) para cada especie, escalamos las variables predictoras (temperatura mínima y máxima, precipitación y fracción lunar) centrando alrededor de la media y dividiendo por dos desviaciones estándar (Schielzeth, 2010). Para cada especie, comparamos 16 modelos aditivos con diferentes combinaciones de variables predictoras, incluyendo un modelo nulo. Identificamos el modelo mejor clasificado con mayor frequencia ($\pi_i$) basado en el criterio de información delta de Akaike ($\Delta$AIC) y el peso de Akaike ($w_i$) tras un remuestreo de 10.000 veces. Para una mejor estimación del modelo mejor clasificado, en cada remuestreo

seleccionamos aleatoriamente sólo 50 de los 61 días de grabaciones y estimamos el porcentaje de veces que se seleccionó cada modelo mejor clasificado ($\pi_i$ ) (Supplemental Data S9). Los análisis fueron conducidos en R, y nuestro código y datos están disponibles en la red (https://github.com/diegryllid).

# Resultados

Nos centramos en siete especies identificadas de ortópteros para nuestro análisis del ensamblaje acústico. Dos especies de grillos (superfamilia Grylloidea): "Flutist" (Cricket1) y Podoscirtinae (Cricket2) y cinco esperanzas (familia Tettigoniidae): *Copiphora colombiae* (Katydid1), *Neoconocephalus brachypterus* (Katydid3)*,* Cocconotini (gen. nov.) (Katydid4), "Sprinkler" (Katydid2), y "Rattler" (Katydid5). Aunque no se cubrieron todas las especies del ensamblaje acústico, estas siete especies fueron las más representativas en términos de actividad acústica (las que se detectaron la mayoría de los días) a lo largo del periodo de muestreo, tal y como se definió tras la anotación manual de las grabaciones.

*Comportamiento de canto de los machos, partición de microhábitats y cantos del ensamblaje*

Detectamos las especies que habitan en diferentes microhábitats por sonotipo *in situ*. En la parte superior del bosque, detectamos a los habitantes del dosel (Cricket1, Katydid2, Katydid5). En el estrato medio, entre los arbustos y las ramas bajas de los árboles (<4m), detectamos a Podoscirtinae (Cricket2). De manera similar, detectamos a Cocconotini gen. nov. (Katydid4) en alturas de ~4m como máximo. Por último, detectamos a Katydid1 y Katydid3 en zonas más abiertas y en arbustos de sucesión temprana. *Cophiphora colombiae* (Katydid1) canta desde debajo de las hojas o en la parte superior de los tallos de los arbustos del sotobosque a unos 2 m de altura, mientras que *N. brachypterus* (Katydid3) siempre canta desde macollas de pasto, y muy cerca del suelo (0,5-1m). Las alturas aquí referidas son estimaciones visuales, por lo que recomendamos la medición directa de las alturas de los individuos para una descripción detallada de la distribución espacial en los estratos del bosque. También detectamos dos comportamientos principales de canto. El primero fue un comportamiento de canto críptico, que consiste en cantar a oculto, observado en dos especies (Cricket2 y Katydid4). La especie de grillo Podoscirtinae (Cricket2) siempre canta desde el interior de hojas de roble enrolladas, mientras que la especie de katydid Cocconotini gen. nov. (Katydid4) canta desde su propia madriguera tallada en troncos de árboles (Cadena-Castañeda, Gómez-Morales, Acevedo-Charry, Benavides-López, *Manuscrito no publicado*). El segundo comportamiento fue el canto expuesto, que consiste en llamar desde lo alto de las hojas y ramas, observado en dos especies de esperanzas: *C. colombiae* (Katydid1) y *N. brachypterus* (Katydid3). No pudimos observar el comportamiento de canto en los habitantes del dosel (Cricket1, Katydid2, Katydid5).

*Actividad acústica diaria*

La actividad acústica diurna varió a lo largo de la noche, reflejando dos estrategias. Todas las esperanzas tienen una estrategia de canto completamente nocturna (Katydid1, Katydid2, Katydid3, Katydid4, Katydid5), aunque "Rattler" (Katydid5) mostró unas pocas detecciones durante el día (Supplemental data S7). Estas cinco especies comienzan a cantar al anochecer (~17:30 hrs); más adelante, tres especies muestran una mayor densidad de detecciones en momentos específicos de la noche (Katydid1: ~20:30, Katydid2: ~01:30, Katydid3: ~20:00) quese pueden observar como el pico de densidad de actividad acústica (Fig. 1). Es importante señalar que estas tres especies siguen activas durante toda la noche, y sus picos de actividad son sutiles (menos de 0,1 de diferencia en la densidad de actividad acústica, si se compara con la media del resto de horas de actividad). Las dos esperanzas restantes (Katydid4, Katydid5) mostraron una actividad acústica relativamente constante durante toda la noche y una disminución más rápida de la actividad desde las 04:00 hasta el amanecer (~06:00). Por otra parte, ambos grillos tienen un horario crepuscular. Podoscirtinae (Cricket2) mostró un horario de canto mucho más restringido, desde las 18:00 hasta las 19:00 con una disminución muy brusca de la actividad. "Flutist" (Cricket1) mostró una disminución más lenta de la actividad, manteniendo un número considerable de detecciones después de las ~21:00. En general, identificamos un momento del coro en el que todos los miembros del ensamblaje cantan al mismo tiempo, desde las 18:00 hasta las 20:00 (Fig. 1).

*Solapamiento de actividad diaria, y espectral*

El análisis por pares de la distribución de la densidad de las especies mostró dos tendencias de solapamiento (Tabla 1). Los grillos tienen menos probabilidad de solaparse con los horarios de canto de las esperanzas (valores de coeficiente bajos <0,6). De ellos, Podoscirtinae (Cricket2) es el más especializado, con un horario de canto muy corto (Fig. 1). Por el contrario, las esperanzas se solapan entre sí en sus periodos de canto (valores de coeficiente altos >0,6).

La frecuencia dominante media fue considerablemente menor para los grillos y mayor para las esperanzas. Mientras que el ancho de banda de los grillos es angosto, el ancho de banda de las esperanzas varió entre las especies, y mostró solapamiento espectral en cuatro especies (Fig. 2). Las especies que utilizaron una mayor frecuencia de canto también incluyeron un mayor ancho de banda (Katydid5, Katydid4, Katydid3), y solapamiento entre ellas. La especie con mayor ancho de banda, *N. brachypterus* (Katydid3), se solapó además con una especie habitante del dosel, Katydid2, que tiene un ancho de banda de frecuencia menor. Aunque la mayoría de las especies tienen componentes armónicos en su canto de llamado (todas excepto Cricket1), como se observó en las grabaciones direccionales (Fig. 2a) con armónicos ultrasónicos en todas las esperanzas excepto Katydid2. (Romer y Lewald, 1992; Hung y Prestwich, 2004). Los armónicos ultrasónicos no se registraron ni analizaron debido a la tasa de muestreo utilizada en nuestra configuración PAM; tampoco se analizaron los armónicos audibles distintos del armónico dominante (el armónico de mayor amplitud), ya que su menor amplitud los hizo indetectables en las grabaciones PAM.

*Respuesta de la actividad acústica a las variables ambientales*

La respuesta de la actividad acústica varió según las especies y las variables ambientales obtenidas por teledetección (Fig. 3, Tabla 2). La actividad acústica de las especies de esperanzas presentó una relación negativa con la fracción lunar para Katydid1 ($\beta = -0{,}28$, $p = 0{,}003$), Katydid2 ($\beta = -0{,}34$, $p < 0{,}001$), Katydid3 ($\beta = -0{,}36$, $p < 0{,}001$), Katydid4 ($\beta = -0{,}13$, $p < 0{,}001$), y Katydid5 ($\beta = -0{,}07$, $p = 0{,}02$). Las dos especies de grillos mostraron una relación negativa sólo con la precipitación (Cricket1: $\beta = -0{,}31$, $p = 0{,}006$; Cricket2: $\beta = -0{,}32$, $p = 0{,}002$) mientras que en las esperanzas la respuesta fue más compleja. Además de con las especies de grillos, la precipitación también estuvo fuertemente relacionada de forma negativa con las actividades acústicas de Katydid1 ($\beta = -0{,}25$, $p = 0{,}02$), y Katydid5 ($\beta = -0{,}06$, $p = 0{,}05$) en menor medida. La temperatura mínima mostró una tendencia positiva tanto para Katydid1 ($\beta = 0{,}26$, $p = 0{,}007$) como para Katydid2 ($\beta = 0{,}21$, $p = 0{,}04$), y la temperatura máxima estuvo significativamente relacionada de forma negativa sólo para Katydid5 ($\beta = -0{,}07$, $p = 0{,}03$). En términos generales, la precipitación diaria explica mejor la actividad acústica de los grillos, mientras que la fracción lunar explica mejor actividad acústica de las esperanzas.

# Discusión

A pesar de que la actividad acústica de los insectos impulsa los paisajes sonoros de los ecosistemas tropicales (Aide et al., 2017), rara vez se identifican con una resolución taxonómica precisa en los estudios ecoacústicos. Utilizamos la huella de señalización acústica de diferentes especies a partir de un muestreo acústico pasivo para caracterizar un ensamblaje de ortópteros. Con el patrón de actividad acústica diaria de cada sonotipo, pudimos muestrear las especies prioritarias y asignar especie taxonómica a cuatro de siete especies. A pesar de las recientes contribuciones al trabajo taxonómico del grupo para Colombia (Cadena-Castañeda et al., 2020, 2021) e investigaciones relevantes sobre la producción y recepción de sonidos (Baker et al., 2019; Celiker, Jonsson y Montealegre-Z, 2020), encontramos novedades taxonómicas en esta comunidad (Cadena-Castañeda, Gómez-Morales, Acevedo-Charry, Benavides-López, *Manuscrito sin publicar*). Además, exploramos la relación de la actividad acústica de cada especie con las variables ambientales provenientes de teledetección por satélite. Sin embargo experimentamos dificultades en la recolección de los habitantes del dosel, para lo cual recomendamos el uso de métodos especializados como la nebulización (Montealegre-Z et al., 2014), trampas de luz (por ejemplo, Symes et al., 2021) o la localización manual especializada (Diwakar & Balakrishnan, 2007b) para futuros estudios. Dado que los estudios sobre los efectos del medio ambiente en las comunidades acústicas de insectos son todavía escasos, nuestro estudio es un precedente importante, y sirve como un buen ejemplo de cómo los datos de teledetección por satélite pueden ser utilizados junto con esquemas de monitoreo acústico en áreas con poco acceso a métodos de medición ambiental local, como las estaciones meteorológicas.

*Especies audibles representativas*

Las especies en las que nos enfocamos representan los ortópteros nocturnos audibles más comunes en el lugar de muestreo. Este enfoque centrado en las especies conspicuas de baja frecuencia (espectro audible) tiene tanto beneficios como desafíos. Al centrarnos en las especies más comunes: Por un lado, pudimos describir la identidad de los sonotipos del ensamblaje, así como la tendencia general de los comportamientos de canto que dominan la comunidad. Sin embargo, se dejaron de lado otras especies que son raras o indetectables por nuestros métodos. Las especies diurnas (saltamontes en su mayoría), por ejemplo, tuvieron una detectabilidad relativamente baja en nuestro conjunto de datos, siendo apenas detectables, lo que confirma las observaciones anteriores para el grupo en los bosques tropicales (Diwakar & Balakrishnan, 2007a). Para estudios cuyo objetivo sea describir ampliamente una comunidad acústica, o analizar las interacciones detalladas entre especies, recomendamos utilizar un muestreo más amplio tanto en espacio (más unidades de grabación) como en tiempo (períodos de monitoreo más largos) en conjunto con una anotación más intensiva de las grabaciones. Por ejemplo, al muestrear en frecuencias ultrasónicas para una adecuada detección de algunas especies de esperanzas neotropicales. El uso de múltiples unidades de grabación también puede mejorar la detectabilidad de especies de canto de baja amplitud, que pueden haber sido infra detectadas en nuestro estudio. A pesar de ello, y dada su alta actividad y detectabilidad, combinada con nuestra capacidad para asignar la resolución taxonómica de la mayoría de las especies; consideramos que las especies representativas aquí consideradas son adecuadas y suficientes para analizar las relaciones de especies individuales con factores ambientales, con propósitos de monitoreo ecoacústico. Por tanto, recomendamos este enfoque de priorización de especies para estudios ambientales con limitaciones metodológicas o temporales.

*Solapamiento temporal y espectral*

Observamos que cuanto más alta es la frecuencia dominante del canto de llamado (Fig. 2), más amplio parece ser el rango de actividad acústica diurna (Fig. 1; Figura Suplementaria 1). Sin embargo, este patrón debe ser estudiado en detalle en futuras investigaciones, con énfasis en los mecanismos subyacentes y contemplando la competencia interespecífica, las limitaciones morfológicas, y/o las relaciones depredador-presa. Mientras que las especies de grillos mostraron llamados de baja frecuencia, agrupados estrechamente en el crepúsculo (Fig. 1, línea punteada), las esperanzas dispersaron sus llamados a lo largo de la noche. Además, las esperanzas con cantos de mediana frecuencia (Fig. 1, líneas discontinuas; Katydid1, Katydid2, Katydid3) mostraron claros picos de actividad, mientras que las de cantos de alta frecuencia (Fig. 1, líneas sólidas; Katydid4, Katydid5) mantuvieron niveles de actividad constantes durante toda la noche. En estudios anteriores se encontró una relación negativa entre la duración de la señal de llamado y la tasa de señalización diaria en esperanzas neotropicales como resultado de compromisos ("trade-off's" en inglés) acústicos (Symes et al., 2021), nuestros datos sugieren que también podría existir una relación positiva entre la frecuencia dominante y la actividad acústica diaria.

Además, a pesar de que no medimos el tamaño del cuerpo o el tamaño específico y las propiedades de las estructuras sonoras productoras de las especies de nuestro ensamblaje, los grillos eran en general más pequeños que las especies de esperanzas, por lo que nuestra observación podría ser contraria a las predicciones de la hipótesis de adaptación morfológica (Farina, 2014), demostrada anteriormente en aves (Wallschläger, 1980), mamíferos (Fletcher, 2004), y ranas (Boeckle, Preininger y Hödl, 2009). Dicha hipótesis podría no ser siquiera aplicable a los ortópteros, dado que su forma de producir sonidos (estridulación elitral) es completamente diferente. Por ejemplo, Godthi et al. (2022) encontraron una relación entre el tamaño y las propiedades del aparato estridulatorio con la frecuencia acústica, independiente del tamaño del cuerpo. Un examen más detallado de nuestros especímenes voucher en el IAvH-E podría ser útil para probar las hipótesis morfológicas. Por último, estudios anteriores han demostrado que los depredadores nocturnos, como los murciélagos, escuchan y seleccionan a sus presas basándose en ciertas propiedades de la señal, incluyendo la frecuencia dominante, y la exposición varía entre las especies presentes en una comunidad (Falk et al., 2015). Entonces, en este caso, las especies de menor frecuencia de pico en este ensamblaje podrían estar bajo mayores presiones de depredación, y responder concentrando su canto en períodos de tiempo más cortos para disminuir ese riesgo (Farina & Ceraulo, 2017). Mas estudios experimentales sobre la agrupación de la actividad acústica como respuesta a predación en insectos son necesarios.

Además de la agregación en el tiempo, existe una clara preferencia por las horas del crepúsculo por parte de los grillos, que puede explicarse porque es el momento del día en el que los depredadores diurnos ya están bajando su actividad, mientras que los depredadores nocturnos aún no están en su pico (Jain et al., 2014). Además, es el momento en el que todas las especies de esperanzas inician su actividad también, por lo que el canto interespecífico simultáneo puede reducir el riesgo individual al "confundir" al depredador que ahora tiene muchas opciones disponibles (Farina & Ceraulo, 2017). Anteriormente nos referimos a este período como el "coro del crepúsculo", y muchos otros factores pueden estar influyendo en este fenómeno. Además, el solapamiento temporal relativamente alto de las esperanzas (Tabla 1; Fig. 1) sugiere que las esperanzas también están agregando su actividad acústica en el tiempo, sólo que de una manera diferente: concentrando su actividad más tarde y extendiéndola durante toda la noche. Mantener un canto continuo, pero prolongando su emisión durante varias horas también tiene sus inconvenientes. Estos requieren adaptaciones en respuesta a los depredadores visuales, como se discutirá más adelante. En trabajos anteriores se ha descrito cómo la agregación espacial de esperanzas puede disminuir la eficacia de las capturas por parte de murciélagos (Prakash et al., 2021). Aunque no medimos la relación entre la frecuencia acústica y la duración de la actividad de llamada diurna, nuestro análisis preliminar parece sugerir que las especies con frecuencias dominantes más bajas tienden a llamar durante períodos más cortos (por ejemplo, Cricket1 vs Katydid5; Figura Suplementaria 1), y viceversa. Para confirmar esta "relación frecuencia-actividad acústica" se requieren trabajos que incluyan más taxones y lugares de muestreo, y que

tengan en cuenta los mecanismos que subyacen al coro del crepúsculo junto con las interacciones acústicas con los depredadores. Adicionalmente, no tuvimos en cuenta las limitaciones filogenéticas o evolutivas al contrastar estos dos grupos de insectos con limitantes muy distintos en la frecuencia de sus cantos, dejando abierto ese tema de investigación para entender mejor el ensamblaje acústico de los ortópteros.

Aunque cuatro especies muestran un solapamiento espectral (Fig. 2), éstas podrían tener diferentes preferencias de microhábitat: Katydid2 parece habitar en el dosel, mientras que Katydid3 se encontró siempre en trozos de hierba a nivel del suelo. Aunque las observaciones de microhábitat se basan en un muestreo oportunista, nuestros resultados proporcionan una aproximación para intentar comprender el solapamiento espectral y temporal de algunas especies. Las especies Katydid4 y Katydid5, que se solapan tanto en tiempo como en frecuencia, habitan el sotobosque y el dosel respectivamente; y pueden estar evitando el enmascaramiento por la atenuación diferencial a diferentes alturas (Ellinger & Hödl, 2003). Sin embargo, otros estudios han encontrado pruebas contradictorias (Jain & Balakrishnan, 2012). Es necesario seguir investigando para comprobar esta hipótesis, ya que el efecto de la estratificación del bosque sobre la interferencia de las señales en los insectos sigue sin estar claro (Schmidt & Balakrishnan, 2015). Además, la medición de características accústicas temporales de menor escala podría ayudar a confirmar si se está evadiendo el enmascaramiento temporal a escalas más pequeñas (Symes et al., 2021), ya que las frecuencias de pulsos, y la duración de las sílabas de los Katydid4 y Katydid5 parecen ser muy diferentes.

*Efectos de los factores ambientales y datos de teledetección*

Las especies de esperanzas disminuyen su actividad acústica entre 0,70 y 0,93 veces con un aumento de una unidad de la fracción lunar iluminada (desde luna nueva en 0,0 hasta luna llena en 1,0). La relación negativa encontrada entre la fracción lunar y la actividad acústica de las especies de esperanzas (Tabla 1, Fig. 3) corrobora los hallazgos de estudios anteriores. Por ejemplo, mayor luz lunar llevó a una respuesta de evasión de los depredadores visuales, ya fuera disminuyendo su actividad general (Lang et al., 2006) o aumentando el uso de canales de comunicación alternativos, como la tremulación (Römer, Lang y Hartbauer, 2010) como parte del repertorio de adaptaciones de las esperanzas a la depredación por parte de murciélagos (ter Hofstede et al., 2017). La respuesta a la luz de luna es probablemente consecuencia de la ya mencionada agrupación de los cantos en un tiempo más amplio; en contraste con los grillos, que de hecho no mostraron ninguna relación con la luz de luna (Tabla 2), quizás porque durante el crepúsculo todavía hay suficiente luz solar, como para que la luz de la luna no marque ninguna diferencia.

En cuanto a la precipitación, un grillo y dos especies de esperanzas disminuyen su actividad acústica entre 0,73 y 0,94 veces con un aumento unitario de la precipitación (mm por día). Este efecto negativo expresándose principalmente sobre la actividad acústica de las dos especies de

grillos (Cricket1 y Cricket2; Fig. 3) confirma observaciones previas (Alexander & Meral, 1967) y las nuestras propias: siempre que llovía durante el coro crepuscular, los grillos a duras penas cantaban. Aunque el ruido de la lluvia puede haber afectado a la detectabilidad en los días de lluvia, nuestra revisión manual de las grabaciones y las observaciones en el campo nos dan confianza en que las detecciones del modelo reflejan la actividad acústica incluso durante los días de lluvia. Aun así, recomendamos el uso de modelos de detección acústica que tengan en cuenta la detección de lluvia, como forma de medir el impacto de la lluvia sobre la detección de ortópteros en futuros estudios. Las esperanzas no mostraron la misma relación con la precipitación diaria, excepto Katydid1 y Katydid5, que mostraron respuesta en conjunto a otras covariables. Sin embargo, algo que hay que tener en cuenta es que dado que el período de llamado de los grillos es más corto, tienen más posibilidades de ser interrumpidos por la lluvia como se ha observado en otras especies (Alexander & Meral, 1967; Franklin et al., 2009); a la vez que los intervalos de llamado más amplios de las esperanzas pueden haber mejorado su detectabilidad incluso después de breves períodos de lluvia durante la noche. Nuestra opinión es que la lluvia puede afectar al canto de los ortópteros a una escala temporal más fina en adición a los efectos diarios acumulados, a diferencia de en la luz de luna, dado que la precipitación diaria puede estar distribuida tanto forma extendida durante el día, como concentrada en períodos cortos. Además, otros factores externos como el viento (Velilla et al., 2020) o el ruido ultrasónico de fondo (Römer & Holderied, 2020) pueden afectar también al comportamiento de señalización de las especies de esperanzas. Una comparación directa de la precipitación medida localmente con datos de teledetección por satélite, y su efecto en la actividad acústica de una comunidad puede ser útil para confirmar esta relación.

El análisis de la temperatura sugiere una relación negativa (disminución de 0,93 veces por unidad de incremento) con la temperatura máxima diaria sólo para Katydid5, y una relación positiva con la temperatura mínima para Katydid1 (aumento de 1,30 veces por unidad de incremento) y Katydid2 (aumento de 1,23 por unidad de incremento de temperatura mínima). Las razones subyacentes de esta respuesta aún no se han explorado en detalle, pero el aumento de la señalización de las especies Katydid1 y Katydid2 concuerda con estudios anteriores en otras especies (Franklin et al., 2009) que encontraron un aumento de la señalización durante las noches más cálidas. Ya que la preferencia del microhábitat puede desempeñar un papel importante en la regulación de la temperatura, recomendamos también el uso de mediciones a escala local para seguir explorando la relación de la actividad de llamada con esta variable.

Aunque estudios anteriores han evaluado la precisión de los productos de teledetección satelital aquí utilizados (o similares) en estudios con aplicaciones ambientales (Ej, Palomino-Ángel, Anaya-Acevedo y Botero, 2019), o han revisado su potencial para la modelación de ecosistemas (Pasetto et al., 2018), e incluso en estudios PAM (Elise et al., 2022). Ninguno hasta donde sabemos, ha evaluado aún la aplicabilidad de datos de teledetección para responder preguntas de monitoreo en comunidades locales. Recomendamos comparar los datos de las estaciones

meteorológicas locales con los datos de teledetección por satélite, en el contexto de los programas de monitoreo biológico local para evaluar el alcance de la aplicabilidad de este enfoque.

## Conclusiones

Los datos astronómicos de la luz de la luna y de la precipitación por teledetección satelital pueden explicar la actividad acústica de las esperanzas y los grillos en un ensamblaje de ortópteros, y su uso puede ser beneficioso para estudios con limitaciones geográficas, financieras o de otro tipo. Aun así, consideramos que es necesario un análisis más profundo que incluya múltiples puntos de muestreo antes de generalizar los patrones observados aquí, dada la pequeña escala temporal y espacial del presente estudio. Los efectos de los pequeños cambios en los factores ambientales sobre la actividad acústica de las especies observados en nuestro estudio sugieren que los ortópteros podrían ser un indicador clave del cambio ambiental. El modo en que esos cambios podrían extrapolarse a la estacionalidad anual y a otros niveles tróficos suponen un tema de estudio potencial que permitirá comprender mejor las comunidades acústicas de todo el mundo. Por último, el seguimiento acústico de los ortópteros tiene un gran potencial para las evaluaciones ambientales, además de ayudar a responder preguntas ecológicas, y enriquecer las descripciones taxonómicas de biotas poco estudiadas.

## Agradecimientos

A nuestro difunto maestro Germán Amat García por su motivación y apoyo, esperamos haberle hecho sentir orgulloso. Agradecemos a Beatriz Salgado, Paula Caycedo, Oscar Cadena-Castañeda, y Jose Luis Benavides-López por contribuir con ideas y perspectivas en las primeras etapas de este proyecto y las colaboraciones en curso. Agradecemos a Rainforest Connection-RFCx por permitirnos utilizar la plataforma ARBIMON con fines académicos sin pago alguno en ese entonces. Recibimos la motivación para trabajar en la Reserva Natural Los Tucanes por parte de Fernando y Pablo Forero, y sus familias, así como de Zuania Colón-Piñeiro durante su tiempo de coordinación de las actividades de investigación en la reserva. Además, agradecemos los numerosos comentarios constructivos sobre el manuscrito de Zuania Colón-Piñeiro, Sarah McGrath-Blaser, Laurel Symes y otros dos revisores anónimos. También agradecemos a David Gray por sus comentarios adicionales y por alentar esta presentación. Los datos de precipitación utilizados en este trabajo se descargaron del sistema de datos en línea Giovanni, desarrollado y mantenido por el GES DISC de la NASA.

## Referencias

Acevedo-Charry O, Ospina AM, Buitrago-Cardona A, Borja-Acosta KG. 2021. Recomendaciones para registrar y almacenar señales acústicas como especímenes digitales en Colombia. *Biodiversidad en la Práctica* 6:e932.

Aide TM, Corrada-Bravo C, Campos-Cerqueira M, Milan C, Vega G, Alvarez R. 2013. Real-time bioacoustics monitoring and automated species identification. *PeerJ* 1:e103. DOI: 10.7717/peerj.103.

Aide T, Hernández-Serna A, Campos-Cerqueira M, Acevedo-Charry O, Deichmann J. 2017. Species Richness (of Insects) Drives the Use of Acoustic Space in the Tropics. *Remote Sensing* 9:1096. DOI: 10.3390/rs9111096.

Alexander R, Meral G. 1967. Seasonal and Daily Chirping Cycles in the Northern Spring and Fall Field Crickets, Gryllus Veletis and G. Pennsylvanicus. *The Ohio Journal of Science* 67:200–209.

arbimon.rfcx.org.Rainforest Connection Arbimon. *Available at https://arbimon.rfcx.org/*

Baker E, Chesmore D. 2020. Standardisation of bioacoustic terminology for insects. *Biodiversity Data Journal* 8:e54222. DOI: 10.3897/BDJ.8.e54222.

Baker AA, Jonsson T, Aldridge S, Montealegre-Z F. 2019. Complex wing motion during stridulation in the katydid Nastonotus foreli (Orthoptera: Tettigoniidae: Pseudophyllinae). *Journal of Insect Physiology* 114:100–108. DOI: 10.1016/j.jinsphys.2019.03.005.

Blumstein DT, Mennill DJ, Clemins P, Girod L, Yao K, Patricelli G, Deppe JL, Krakauer AH, Clark C, Cortopassi KA, Hanser SF, McCowan B, Ali AM, Kirschel ANG. 2011. Acoustic monitoring in terrestrial environments using microphone arrays: applications, technological considerations and prospectus. *Journal of Applied Ecology* 48:758–767. DOI: 10.1111/j.1365-2664.2011.01993.x.

Boeckle M, Preininger D, Hödl W. 2009. Communication in Noisy Environments i: Acoustic Signals of Staurois Latopalmatus Boulenger 1887. *Herpetologica* 65:154–165. DOI: 10.1655/07-071R1.1.

Bolker B. 2007. *Ecology Ecological Models and Data in R*. Princeton and Oxford: Princeton University Press. DOI: 10.1086/644667.

Bradbury JW, Vehrencamp SL. 2011. *Principles of Animal Communication*. Sunderland, MA: Sinauer Associates, Inc.

Bruni A, Mennill DJ, Foote JR. 2014. Dawn chorus start time variation in a temperate bird community: relationships with seasonality, weather, and ambient light. *Journal of Ornithology* 155:877–890. DOI: 10.1007/s10336-014-1071-7.

Buellesbach J, Cash E, Schmitt T. 2018. Communication, Insects. In: Skinner M ed. *Encyclopedia of Reproduction*. Waltham MA: Elsevier, 78–83. DOI: 10.1016/B978-0-12-809633-8.20539-6.

Cadena-Castañeda OJ, Arango Díaz CJ, Grande López VH, Floréz Cárdenas ADP. 2021. Studies on Neotropical crickets: New species and notes on the classification of Field Crickets genera Anurogryllus and Gryllus (Orthoptera: Gryllidae: Gryllinae). *Zootaxa* 4970:515–532. DOI: 10.11646/zootaxa.4970.3.4.

Cadena-Castañeda O, Prieto-Cristancho D, Romero-Betancourt L, Braun H, García García A. 2020. Studies in Neotropical Pseudophyllinae: A new genus of Cocconotini from the Colombian Andes and new subfamily assignment of the Dominican genus Anacaona (Tettigoniidae: Pseudophyllinae: Cocconotini). *Zootaxa* 4885:133–138. DOI: 10.11646/zootaxa.4885.1.9.

Celiker E, Jonsson T, Montealegre-Z F. 2020. On the tympanic membrane impedance of the katydid Copiphora gorgonensis (Insecta: Orthoptera: Tettigoniidae). *The Journal of the Acoustical Society of America* 148:1952–1960. DOI: 10.1121/10.0002119.

Chhaya V, Lahiri S, Jagan MA, Mohan R, Pathaw NA, Krishnan A. 2021. Community Bioacoustics: Studying Acoustic Community Structure for Ecological and Conservation Insights. *Frontiers in Ecology and Evolution* 9:706445. DOI: 10.3389/fevo.2021.706445.

Climate-Data.org. 2019.Climate Gachantivá (Colombia). *Available at https://en.climate-data.org/south-america/colombia/boyaca/gachantiva-44435/*

Cribari-Neto F, Zeileis A. 2010. Beta Regression in R. *Journal of Statistical Software* 34. DOI: 10.18637/jss.v034.i02.

Deichmann JL, Acevedo-Charry O, Barclay L, Burivalova Z, Campos-Cerqueira M, D'Horta F, Game ET, Gottesman BL, Hart PJ, Kalan AK, Linke S, Nascimento L Do, Pijanowski B, Staaterman E, Mitchell Aide T. 2018. It's time to listen: there is much to be learned from the sounds of tropical ecosystems. *Biotropica* 50:713–718. DOI: 10.1111/btp.12593.

Diwakar S, Balakrishnan R. 2007a. The assemblage of acoustically communicating crickets of a tropical evergreen forest in Southern India: call diversity and diel calling patterns. *Bioacoustics* 16:113–135. DOI: 10.1080/09524622.2007.9753571.

Diwakar S, Balakrishnan R. 2007b. Vertical stratification in an acoustically communicating ensiferan assemblage of a tropical evergreen forest in southern India. *Journal of Tropical Ecology* 23:479–486. DOI: 10.1017/S0266467407004208.

Drewry GE, Rand AS. 1983. Characteristics of an Acoustic Community : Puerto Rican Frogs of the Genus Eleutherodactylus. *Copeia* 1983:941–953. DOI: https://doi.org/1445095.

Ellinger N, Hödl W. 2003. Habitat acoustics of a neotropical lowland rainforest. *Bioacoustics* 13:297–321. DOI: 10.1080/09524622.2003.9753503.

Elise, S, Guilhaumon F, Mou-Tham G, Urbina-Barreto I, Vigliola L, Kulbicki M, Bruggemann JH. 2022. Combining Passive Acoustics and Environmental Data for Scaling Up Ecosystem Monitoring: A Test on Coral Reef Fishes. *Remote Sensing* 14(10): 2394.

Falk JJ, ter Hofstede HM, Jones PL, Dixon MM, Faure PA, Kalko EK V., Page RA. 2015. Sensory-based niche partitioning in a multiple predator–multiple prey community. *Proceedings of the Royal Society B: Biological Sciences* 282:20150520. DOI: 10.1098/rspb.2015.0520.

Farina A. 2014. *Soundscape Ecology*. Dordrecht: Springer Netherlands. DOI: 10.1007/978-94-007-7374-5.

Farina A, Ceraulo M. 2017. *Ecoacoustics: The ecological role of sounds*. Oxford, UK: John Wiley and Sons.

Ferreira LM, Oliveira EG, Lopes LC, Brito MR, Baumgarten J, Rodrigues FH, Sousa-Lima RS. 2018. What do insects, anurans, birds, and mammals have to say about soundscape indices in a tropical savanna. *Journal of Ecoacoustics* 2:#PVH6YZ. DOI: 10.22261/jea.pvh6yz.

Fletcher NH. 2004. A simple frequency-scaling rule for animal communication. *The Journal of the Acoustical Society of America* 115:2334–2338. DOI: 10.1121/1.1694997.

Franklin M, Droege S, Dawson D, Royle JA. 2009. Nightly and Seasonal Patterns of Calling in Common True Katydids (Orthoptera: Tettigoniidae: Pterophylla camellifolia ). *Journal of Orthoptera Research* 18:15–18. DOI: 10.1665/034.018.0108.

Gasc A, Anso J, Sueur J, Jourdan H, Desutter-Grandcolas L. 2018. Cricket calling communities as an indicator of the invasive ant Wasmannia auropunctata in an insular biodiversity hotspot. *Biological Invasions* 20:1099–1111. DOI: 10.1007/s10530-017-1612-0.

Godthi V, Balakrishnan R, Pratap R. 2022. The mechanics of acoustic signal evolution in field crickets. *Journal of Experimental Biology* 225(Suppl_1): jeb243374.

Gottesman BL, Olson JC, Yang S, Acevedo-Charry O, Francomano D, Martinez FA, Appeldoorn RS, Mason DM, Weil E, Pijanowski BC. 2021. What does resilience sound like? Coral reef and dry

forest acoustic communities respond differently to Hurricane Maria. *Ecological Indicators* 126:107635. DOI: 10.1016/j.ecolind.2021.107635.

Grimaldi D, Engel MS. 2005. *Evolution of the Insects*. New York, NY: Cambridge University Press.

Heinicke S, Kalan AK, Wagner OJJ, Mundry R, Lukashevich H, Kühl HS. 2015. Assessing the performance of a semi-automated acoustic monitoring system for primates. *Methods in Ecology and Evolution* 6:753–763. DOI: 10.1111/2041-210X.12384.

ter Hofstede H, Voigt-Heucke S, Lang A, Römer H, Page R, Faure P, Dechmann D. 2017. Revisiting adaptations of neotropical katydids (Orthoptera: Tettigoniidae) to gleaning bat predation. *Neotropical Biodiversity* 3:41–49. DOI: 10.1080/23766808.2016.1272314.

Huffman GJ, Stocker DT, Bolvin EJ, Nelkin JT. 2022. GPM IMERG Final Precipitation L3 1 day 0.1 degree x 0.1 degree V06. DOI: 10.5067/GPM/IMERGDF/DAY/06.

Hung YP, Prestwich KN. 2004. Is significant acoustic energy found in the audible and ultrasonic harmonics in cricket calling songs? *Journal of Orthoptera Research* 13:231–238. DOI: https://doi.org/10.1665/1082-6467(2004)013[0231:ISAEFI]2.0.CO;2.

Jain M, Balakrishnan R. 2012. Does acoustic adaptation drive vertical stratification? A test in a tropical cricket assemblage. *Behavioral Ecology* 23:343–354. DOI: 10.1093/beheco/arr191.

Jain M, Diwakar S, Bahuleyan J, Deb R, Balakrishnan R. 2014. A rain forest dusk chorus: cacophony or sounds of silence? *Evolutionary Ecology* 28:1–22. DOI: 10.1007/s10682-013-9658-7.

Jeliazkov A, Bas Y, Kerbiriou C, Julien JF, Penone C, Le Viol I. 2016. Large-scale semi-automated acoustic monitoring allows to detect temporal decline of bush-crickets. *Global Ecology and Conservation* 6:208–218. DOI: 10.1016/j.gecco.2016.02.008.

Krause BL. 1993. The Niche Hypothesis: A Virtual Symphony of Animal Sounds, The Origins of Musical Expression and the Health of Habitats. *The Soundscape Newsletter* 6:6–10.

Lang AB, Kalko EKV, Römer H, Bockholdt C, Dechmann DKN. 2006. Activity levels of bats and katydids in relation to the lunar cycle. *Oecologia* 146:659–666. DOI: 10.1007/s00442-005-0131-3.

Lapp S, Wu T, Richards-Zawacki C, Voyles J, Rodriguez KM, Shamon H, Kitzes J. 2021. Automated detection of frog calls and choruses by pulse repetition rate. *Conservation Biology* 35(5): 1659-1668.

McGeoch MA. 2007. Insects and Bioindication. In: Stewart AJA, New TR, Lewis OT eds. *Insect conservation biology*. Wallingford: CABI, 144–174.

Montealegre-Z F, Sarria FA, Pimienta MC, Mason AC. 2014. Lack of correlation between vertical distribution and carrier frequency, and preference for open spaces in arboreal katydids that use extreme ultrasound, in Gorgona, Colombia (Orthoptera: Tettigoniidae). *Revista de Biología Tropical* 62:289. DOI: 10.15517/rbt.v62i0.16342.

ocenaudio Team. 2015. ocenaudio.

Ospina OE, Villanueva-Rivera LJ, Corrada-Bravo CJ, Aide TM. 2013. Variable response of anuran calling activity to daily precipitation and temperature: implications for climate change. *Ecosphere* 4:art47. DOI: 10.1890/ES12-00258.1.

Palomino-Ángel S, Anaya-Acevedo JA, Botero BA. 2019. Evaluation of 3B42V7 and IMERG daily-precipitation products for a very high-precipitation region in northwestern South America. *Atmospheric Research* 217:37–48. DOI: 10.1016/j.atmosres.2018.10.012.

Pasetto D, Arenas-Castro S, Bustamante J, Casagrandi R, Chrysoulakis N, Cord AF, Dittrich A, Domingo-Marimon C, El Serafy G, Karnieli A, Kordelas GA, Manakos I, Mari L, Monteiro A,

Palazzi E, Poursanidis D, Rinaldo A, Terzago S, Ziemba A, Ziv G. 2018. Integration of satellite remote sensing data in ecosystem modelling at local scales: Practices and trends. *Methods in Ecology and Evolution* 9:1810–1821. DOI: 10.1111/2041-210X.13018.

Pérez-Granados C, Schuchmann K-L, Marques MI. 2022. Addicted to the moon: vocal output and diel pattern of vocal activity in two Neotropical nightjars is related to moon phase. *Ethology Ecology & Evolution* 34:66–81. DOI: 10.1080/03949370.2021.1886182.

Pettorelli N, Laurance WF, O'Brien TG, Wegmann M, Nagendra H, Turner W. 2014. Satellite remote sensing for applied ecologists: opportunities and challenges. *Journal of Applied Ecology* 51:839–848. DOI: 10.1111/1365-2664.12261.

Prakash H, Greif S, Yovel Y, Balakrishnan R. 2021. Acoustically eavesdropping bat predators take longer to capture katydid prey signalling in aggregation. *Journal of Experimental Biology* 224:1–9. DOI: 10.1242/jeb.233262.

QGIS Development Team. 2022. QGIS: qgis.org.

Ridout MS, Linkie M. 2009. Estimating overlap of daily activity patterns from camera trap data. *Journal of Agricultural, Biological, and Environmental Statistics* 14:322–337. DOI: 10.1198/jabes.2009.08038.

Riede K. 1996. Diversity of sound-producing insects in a Bornean lowland rain forest. :77–84. DOI: 10.1007/978-94-009-1685-2_8.

Riede K. 1998. Acoustic monitoring of Orthoptera and its potential for conservation. *Journal of Insect Conservation* 2:217–223. DOI: 10.1023/a:1009695813606.

Riede K. 2018. Acoustic profiling of Orthoptera: present state and future needs. *Journal of Orthoptera Research* 27:203–215. DOI: 10.3897/jor.27.23700.

Römer H, Holderied M. 2020. Decision making in the face of a deadly predator: high-amplitude behavioural thresholds can be adaptive for rainforest crickets under high background noise levels. *Philosophical Transactions of the Royal Society B: Biological Sciences* 375:20190471. DOI: 10.1098/rstb.2019.0471.

Römer H, Lang A, Hartbauer M. 2010. The Signaller's Dilemma: A Cost–Benefit Analysis of Public and Private Communication. *PLoS ONE* 5:e13325. DOI: 10.1371/journal.pone.0013325.

Römer H, Lewald J. 1992. High-frequency sound transmission in natural habitats: implications for the evolution of insect acoustic communication. *Behavioral Ecology and Sociobiology* 29:437–444. DOI: 10.1007/BF00170174.

Schielzeth H. 2010. Simple means to improve the interpretability of regression coefficients. *Methods in Ecology and Evolution* 1:103–113. DOI: 10.1111/j.2041-210X.2010.00012.x.

Schmidt AKD, Balakrishnan R. 2015. Ecology of acoustic signalling and the problem of masking interference in insects. *Journal of Comparative Physiology A* 201:133–142. DOI: 10.1007/s00359-014-0955-6.

Schmidt AKD, Römer H, Riede K. 2013. Spectral niche segregation and community organization in a tropical cricket assemblage. *Behavioral Ecology* 24:470–480. DOI: 10.1093/beheco/ars187.

Smithson M, Verkuilen J. 2006. A better lemon squeezer? Maximum-likelihood regression with beta-distributed dependent variables. *Psychological Methods* 11:54–71. DOI: 10.1037/1082-989X.11.1.54.

Steinfath E, Palacios-Muñoz A, Rottschäfer JR, Yuezak D, Clemens J. (2021). Fast and accurate annotation of acoustic signals with deep neural networks. *Elife* 10: e68837.

Sugai LSM, Desjonquères C, Silva TSF, Llusia D. 2020. A roadmap for survey designs in terrestrial acoustic monitoring. *Remote Sensing in Ecology and Conservation* 6:220–235. DOI: 10.1002/rse2.131.

Sugai LSM, Silva TSF, Ribeiro JW, Llusia D. 2019. Terrestrial Passive Acoustic Monitoring: Review and Perspectives. *BioScience* 69:15–25. DOI: 10.1093/biosci/biy147.

Symes LB, Robillard T, Martinson SJ, Dong J, Kernan CE, Miller CR, Ter Hofstede HM. 2021. Daily Signaling Rate and the Duration of Sound per Signal are Negatively Related in Neotropical Forest Katydids. In: *Integrative and Comparative Biology*. Oxford University Press, 887–899. DOI: 10.1093/icb/icab138.

Tan MK, Malem J, Legendre F, Dong J, Baroga-Barbecho JB, Yap SA, Wahab R bin HA, Japir R, Chung AYC, Robillard T. 2021. Phylogeny, systematics and evolution of calling songs of the Lebinthini crickets (Orthoptera, Grylloidea, Eneopterinae), with description of two new genera. *Systematic Entomology* 46:1060–1087. DOI: 10.1111/syen.12510.

Thieurmel B, Elmarhraoui A. 2019. suncalc: Compute Sun Position, Sunlight Phases, Moon Position and Lunar Phase.

Tobias JA, Planque R, Cram DL, Seddon N. 2014. Species interactions and the structure of complex communication networks. *Proceedings of the National Academy of Sciences* 111:1020–1025. DOI: 10.1073/pnas.1314337111.

Tovar Garcia JD, Acevedo-Charry O. 2021. Conjunto de datos de monitoreo acústico pasivo en la Reserva Natural Los Yátaros, Gachantivá, Boyacá, Colombia. *Biota Colombiana* 22. DOI: 10.21068/c2021.v22n01a13.

Velilla E, Muñoz M, Quiroga N, Symes L, ter Hofstede HM, Page RA, Simon R, Ellers J, Halfwerk W. 2020. Gone with the wind: Is signal timing in a neotropical katydid an adaptive response to variation in wind-induced vibratory noise? *Behavioral Ecology and Sociobiology* 74:59. DOI: 10.1007/s00265-020-02842-z.

Villanueva-Rivera LJ. 2014. Eleutherodactylus frogs show frequency but no temporal partitioning: implications for the acoustic niche hypothesis. *PeerJ* 2:e496. DOI: 10.7717/peerj.496.

Wallschläger D. 1980. Correlation of song frequency and body weight in passerine birds. *Experientia* 36:412.

Zhang T, Zhou Y, Zhu Z, Li X, Asrar G. 2021. A global seamless 1 km resolution daily land surface temperature dataset (2003 – 2020). DOI: 10.25380/iastate.c.5078492.v3.

Zhang T, Zhou Y, Zhu Z, Li X, Asrar GR. 2022. A global seamless 1 km resolution daily land surface temperature dataset (2003–2020). *Earth System Science Data* 14:651–664. DOI: 10.5194/essd-14-651-2022.
